# Fabrication of Antireflective Nanostructures on a Transmission Grating Surface Using a One-Step Self-Masking Method

**DOI:** 10.3390/nano9020180

**Published:** 2019-02-01

**Authors:** Ting Shao, Feng Tang, Laixi Sun, Xin Ye, Junhui He, Liming Yang, Wanguo Zheng

**Affiliations:** 1 Research Center of Laser Fusion, China Academy of Engineering Physics, Mianyang, Sichuan 621900, China; shaot05@163.com (T.S.); tangfengf3@126.com (F.T.); sunlaixi@126.com (L.S.); yanglcaep@163.com (L.Y.); yehanwin@mail.ustc.edu.cn (W.Z.); 2 Functional Nanomaterials Laboratory, Center for Micro/Nanomaterials and Technology and Key Laboratory of Photochemical Conversion and Optoelectronic Materials, Technical Institute of Physics and Chemistry, Chinese Academy of Sciences, Beijing 100190, China; 3 IFSA (Inertial Fusion Sciences and Applications) Collaborative Innovation Center, Shanghai Jiao Tong University, Shanghai 200240, China

**Keywords:** antireflection, subwavelength structures, self-masking etching, transmission grating

## Abstract

Suppression of Fresnel reflection from diffraction grating surfaces is very important for many optical configurations. In this work, we propose a simple method to fabricate subwavelength structures on fused-silica transmission grating for optical antireflection. The fabrication is a one-step self-masking reaction ion etching (RIE) process without using any masks. According to effective medium theory, random cone-shaped nanopillars which are integrated on the grating surface can act as an antireflective layer. Effects of the nanostructures on the reflection and transmission properties of the grating were investigated through experiments and simulations. The nanostructure surface exhibited excellent antireflection performance, where the reflection of the grating surface was suppressed to zero over a wide range of incident angles. Results also revealed that the etching process can change the duty cycle of the grating, and thus the diffraction orders if there are oblique lateral walls. The simulation results were in good agreement with the experimental ones, which verified our physical comprehension and the corresponding numerical model. The proposed method would offer a low-cost and convenient way to improve the antireflective performance of transmission-diffractive elements.

## 1. Introduction

For decades, significant attention has been paid to the suppression of Fresnel reflection at two-media interfaces for optical applications such as high-performance solar cells, photoelectric detectors, superluminescent diodes and laser optics [1,2,3,4]. Diffractive optical elements (DOEs), which are widely used in many transmission configurations, are also limited by Fresnel reflection. As typical DOEs, diffraction gratings are fundamental optical elements which have wide applications in many devices and measurements, such as refractive index measurement [5] and other measurements with a digital holography approach [6,7,8], where the antireflective properties of the grating would be crucial as they may affect the measurement. Particularly, in high-power laser systems, small Fresnel reflection could cause very large amounts of energy loss. For instance, beam-sampling grating used for diagnostic purposes in the SG series high-power laser facility in China could cause nearly a 4% loss of energy delivered to the fusion target due to Fresnel reflection of the grating surface. Therefore, Fresnel reflection-free optical interfaces are very desirable for DOEs used in high-power laser systems. Over the past years, various antireflection technologies have been developed, which can be divided into two categories; antireflective (AR) coatings with a single-layer film or multi-layer thin-film stacks [9,10,11,12,13] and AR subwavelength structures (SWSs) with graded refractive index profiles [14,15,16].

AR coating is currently the main approach to suppress Fresnel reflection for DOEs [9,17,18,19,20,21,22]. However, inherent limitations of the coating associated with laser-induced damage, absorption-induced contamination, thermal expansion mismatch, and bad mechanical stability always exist for such flat surfaces [23,24]. To obtain broadband antireflection, multi-layer films with specific refractive indexes and thicknesses are required, which largely increases the complexity and difficulty of the fabrication process [18]. In addition, it becomes even more difficult when applying film coating to a grating surface with small grooves and ridges [18,19].

Antireflective SWSs, which are widely applied on flat surfaces [14,15,16] and even curved surfaces [25,26], are another way to suppress Fresnel reflection for DOEs. Since no foreign materials are introduced, this method has remarkable advantages, including a simple fabrication process, good mechanical and environmental durability, high laser-damage resistance, etc. [15,27]. Excellent work has also been done on fabricating SWSs on microstructured arrays to produce biomimetic multifunctional hierarchical nano/micro structures [28,29,30]. However, few reports are available on fabricating SWSs on diffractive grating surfaces. Order-arranged nanospheres have been utilized to fabricate SWSs on a silicon grating surface [31]. Although nearly zero reflection loss was achieved, multiple complicated and time-consuming procedures were contained in the fabrication process. More recently, a simpler method, using reactive ion etching (RIE), was adopted to create random antireflective SWSs on both flat and grating surfaces of fused silica [32]. However, the mask fabrication procedure was still tedious. Additionally, noble metals, such as Au, used for fabricating masks make this technique costly. The self-masking RIE technique, which can create random masks through by-product fluorocarbon during the etching [16], is a promising candidate for fabricating SWSs on grating surfaces. While this approach has been applied efficiently on flat surfaces [15,16], research has been done on the implementation of this technique to grating surfaces [33]. Particularly, no study has reported on the effects of SWSs fabricated by this approach on the antireflection and diffraction properties of the grating.

In this work, we adopt a simple, low-cost, one-step self-masking RIE technique to fabricate SWSs on fused-silica transmission gratings. Compared to the published methods that fabricate SWSs on diffraction gratings [31,32], the present approach is much simpler and cheaper to implement. The antireflection performance of the fabricated SWSs and its influence on the grating’s transmission are investigated experimentally and numerically.

## 2. Results and Discussion

**Figure 1:**
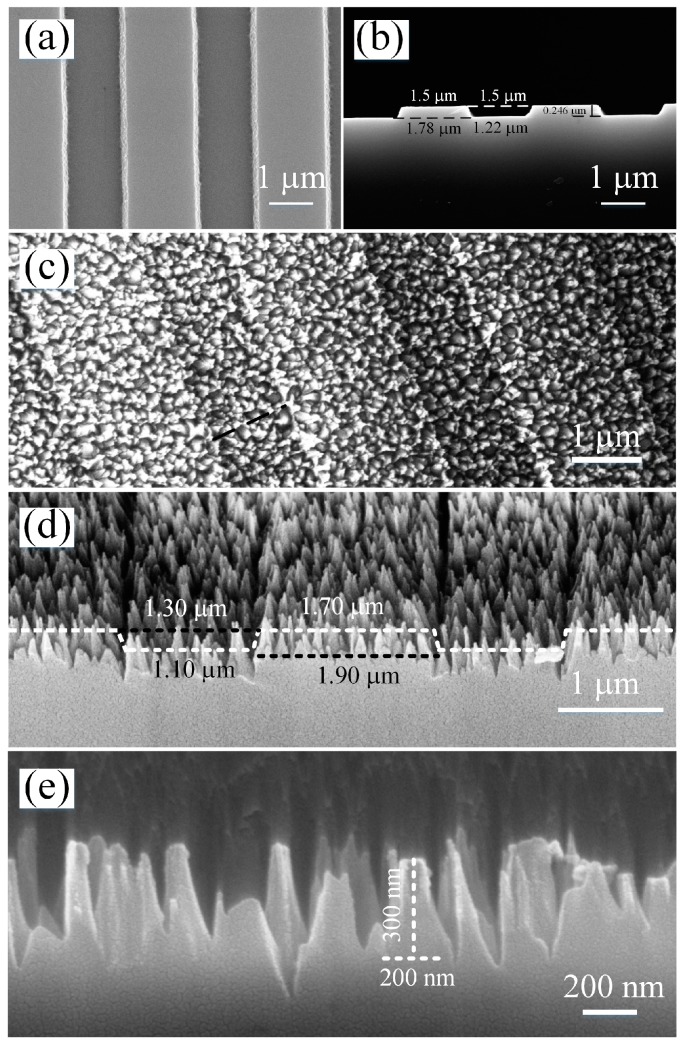
Scanning electron microscope (SEM) images of the grating before and after the cone-shaped subwavelength structures (SWSs) are fabricated. (**a**) Top view and (**b**) Side view of the bare grating. (**c**) Top view and (**d**) oblique view (45°) of the nanostructured grating. (**e**) Side view of the SWSs.

Since the preparation of beam-sampling grating is high-cost and time-consuming, gratings that also have a micro-ridge structure with the size of a few micrometers were used as models to implement the study. The bare gratings were homemade using optical lithography on fused silica substrates with the size of 20 mm × 20 mm × 1 mm. The bare grating had trapezoidal ridges and grooves with a period of 3 μm and a depth of 250 nm, as shown in Figure 1a,b. The duty cycle, which is defined as the ratio of half ridge-width over period, was about 0.55. Before fabricating the SWSs, the bare gratings were cleaned with Micro-90 (a kind of alkalescent cleaning agent). Gas reactants, used for implementing the self-masking RIE, were trifluoromethane (CHF_3_), sulfur hexafluoride (SF_6_), and helium (He). In the etching process, by-product fluorocarbon can be deposited on the grating surface and work as random masks. After 20 minute etching, SWSs were successfully fabricated on the grating surface.

The morphologies of the nanostructured grating observed by scanning electron microscope (SEM) are presented in Figure 1c–e. It can be clearly seen that the grating ridges and grooves were covered with random nanostructures and the ridge-groove profile of the nanostructured grating is unchanged (see the white dotted lines in Figure 1d). Because of the “negative” lateral etching, the duty cycle of the nanostructured grating increases to 0.65. On the ridges and grooves of the nanostructured grating, the randomly-distributed nanostructures have the same appearance of cone-shaped pillars. The SEM image in Figure 1e shows the nanopillars with higher magnification. The cone-shaped nanopillars have an average height of around 300 nm and bottom width of around 200 nm.

**Figure 2:**
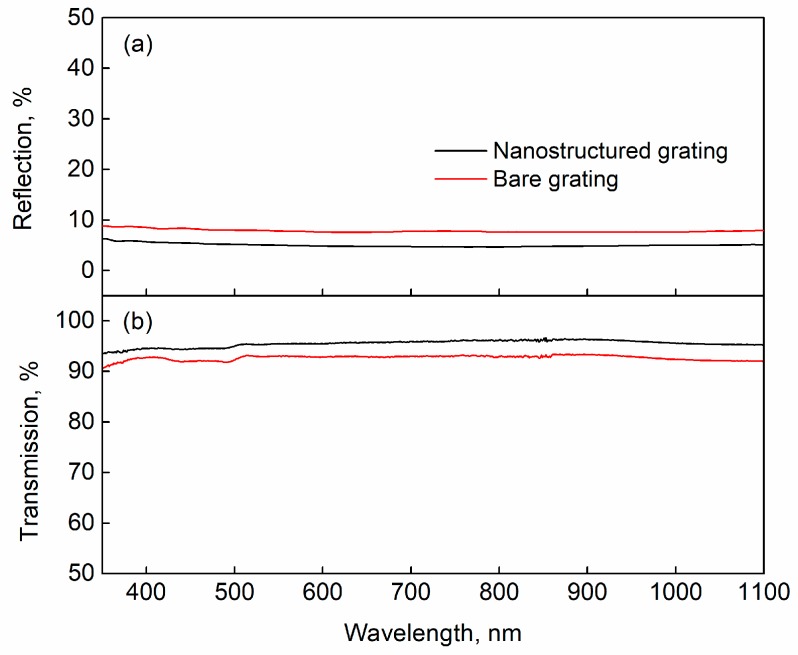
Total (**a**) reflection spectra and (**b**) transmission spectra of the grating before and after the etching measured by the lambda 950 spectrometer equipped with an integrating sphere.

To show the antireflection effects of the SWSs, the total reflection and transmission efficiencies of the grating before and after the etching were measured respectively, as shown in Figure 2. The incident light was injected from the grating’s back side at normal incidence. It is obvious that the gratings with SWSs exhibit excellent antireflection performance over a wide optical bandwidth. At a waveband ranging from 350 nm to 1100 nm, the reflection efficiency of the bare grating was about 8% while the transmission efficiency was about 92%. After the SWSs were fabricated on the grating surface, the reflection efficiency decreased to about 5% while the transmission efficiency increased to about 95%.

**Figure 3:**
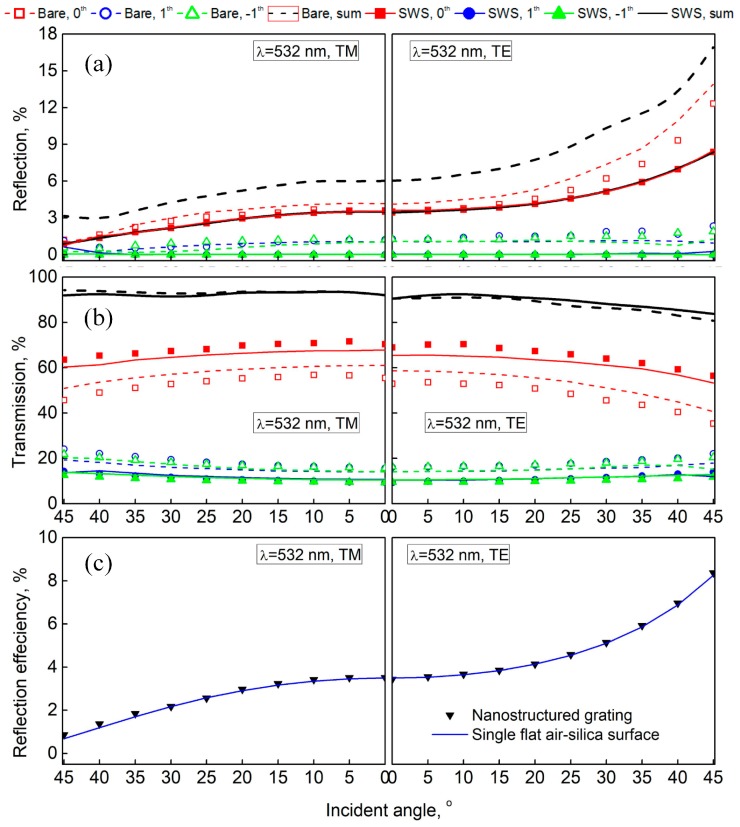
(**a**) Reflection efficiencies and (**b**) transmission–diffraction efficiencies of the bare/nanostructured grating for different diffraction orders as functions of incident angle at the wavelength of 532 nm. The dots present the experimental results, while the colored lines illustrate the simulated ones. The colors (red, green and blue) denote different diffraction orders (0th, −1st and +1st, respectively). Open dots and dashed lines represent the bare grating, while solid dots and solid lines represent the nanostructured grating. The black lines show the total efficiencies of all the reflection or transmission–diffraction orders obtained from the experiments. (**c**) Comparison of the reflection efficiency of a single air–silica interface calculated by finite-difference time-domain (FDTD) and the total reflection efficiency for all the reflection–diffraction orders of the nanostructured grating obtained experimentally.

For each individual diffraction order, the reflection and transmission efficiencies of the grating are presented in Figure 3. The data were obtained using a homemade optical setup, where the incident light with a wavelength of 532 nm was injected from the grating’s back side and a polarized beam splitter (PBS) was used to control the incident polarization (TE or TM). Meanwhile, the numerical calculation of the reflection and transmission efficiencies of the bare/nanostructured grating are also included in Figure 3, which were based on the finite-difference time-domain (FDTD) method. In the FDTD simulations, the bare grating has the same geometrical parameters as those in Figure 1b, and the nanostructured grating had a new duty cycle of 0.65 and randomly-distributed nanocones with a height of 300 nm and bottom width of 200 nm. The refractive indexes of air and fused silica were set to be 1 and 1.46 respectively. More details about the experiments and simulations are given in the Supplementary Materials, where the experimental setup and FDTD model are illustrated in Figures S1 and S2 respectively.

It is obvious from Figure 3 that the simulated results agree well with the experimental ones. Compared to the bare grating, all the reflection–diffraction orders of the nanostructured grating were remarkably suppressed. Particularly, the reflection efficiencies of the ±1st orders were suppressed to nearly zero for all incident angles. Nearly no reflection–diffraction orders of the nanostructured grating could be observed except for the 0th order, which can be inferred from the fact that the total reflection efficiency was the same to that of the 0th order (see the coincidence of the solid black and solid red lines in Figure 3a). Moreover, the light intensity of the 0th order came from the Fresnel reflection of the grating’s back side (i.e., the incident interface), not from the grating surface. It makes us believe that the reflection from the grating surface has been suppressed to zero after the SWSs were fabricated on the grating surface, only leaving the reflection from the grating’s back side. More clear understanding can be obtained by comparing the reflection efficiency of a single air–silica interface calculated by FDTD simulations and the total reflection efficiency of all the reflection–diffraction orders of the nanostructured grating obtained in experiments, as shown in Figure 3c. The two items turn out to be exactly the same as each other, which proves the inference of the complete elimination of reflection from the grating surface with SWSs. According to effective medium theory, we believe that the total suppression of Fresnel reflection from the grating surface is attributed to the gradual change of the effective refractive index between the fused silica substrate and air, which is provided by the cone-shaped nanopillars of the SWSs. For the way in which effective medium theory was adopted herein, please refer to the Appendix A at the end of this paper.

**Figure 4:**
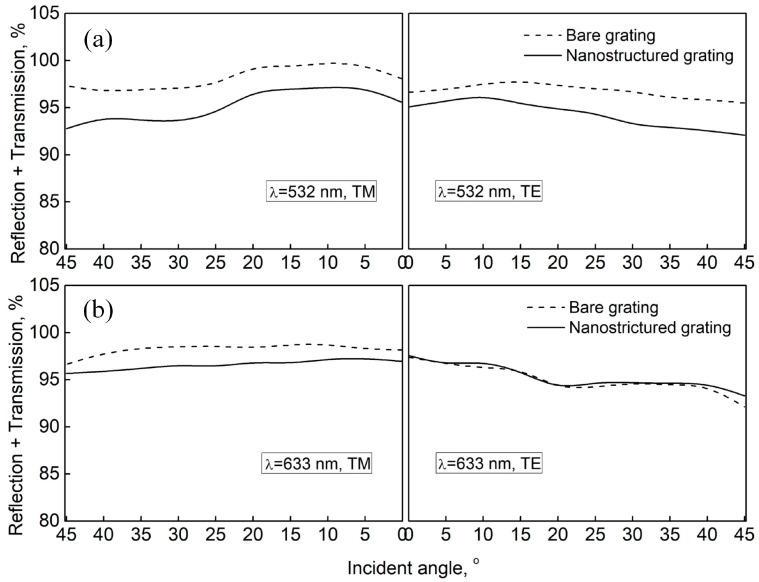
The sum of total reflection and total transmission (*R*_t_ + *T*_t_) of the grating with and without SWSs at a wavelength of (**a**) 532 nm and (**b**) 633 nm.

Since the reflection of the grating was suppressed through the introduction of the SWSs layer, the transmission was consequently improved, as Figure 3b shows. However, the increase of the total transmission efficiency (~3%) was smaller than the decrease of the total reflection efficiency (~8%). Especially, at some large incident angles with TM polarization, the total transmission through the nanostructured gating was even somewhat lower than that of the bare grating. This discrepancy can be more clearly observed in Figure 4a, which gives the sum of the total reflection and total transmission (*R*_t_ + *T*_t_) of the grating with and without SWSs at a wavelength of 532 nm. We speculated that the decrease of *R*_t_ + *T*_t_ was probably due to the scattering of light on the disordered SWSs since the size and period of the SWSs in this study were not much smaller than the wavelength of the incident light. Based on this speculation, we believed that the decline of *R*_t_ + *T*_t_ would become weak when the light wavelength was increased. To verify it, the reflection and transmission of the same grating at a larger wavelength were measured with the same experimental setup. In this measurement, a 632.8 nm incident light was used. As shown in Figure 4b, as the wavelength increased from 532 nm to 632.8 nm, the decrease of *R*_t_ + *T*_t_ is much weaker than that at the wavelength of 532 nm, which verified our speculation above. So, the scattering of light can be further suppressed or even totally avoided with smaller nanostructures and longer working wavelengths, leading to further transmission improvement of the nanostructured interface.

From the efficiencies of each transmission–diffraction order in Figure 3b, we also noticed that the transmission efficiency of the 0th order increased significantly while the efficiencies of the ±1st orders decreased a little compared to the bare grating. This is likely attributed to the change of the grating’s duty cycle due to RIE treatment, which can be identified by comparing Figure 1c with Figure 1a and Figure 1d with Figure 1b. In fact, the duty cycle of the grating increased from 0.55 to 0.65 after the SWSs were fabricated on the grating surface. This result suggests that the etching was not unidirectional and had “negative” lateral etching. This is because the oblique trapezoidal lateral walls made the etching velocity vector oblique. So, the variations of etching rate depending on the topography should be taken into consideration when applying self-masking RIE on the non-flat surface for fabricating antireflective SWSs.

**Figure 5:**
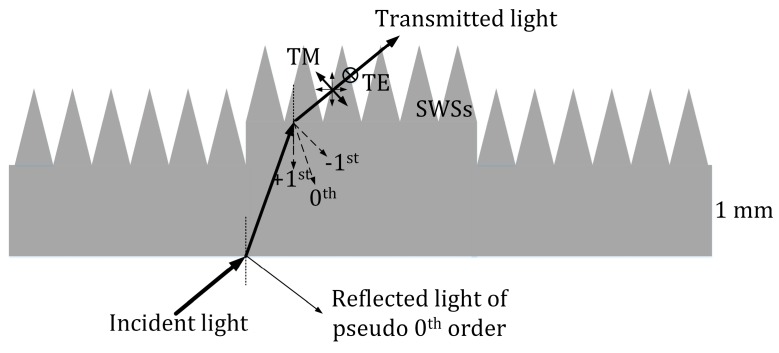
Schematics of the propagation of light in the nanostructured grating in the experiment. The “reflected light of pseudo 0th order” denoted in this figure is composed of the reflected light from the flat air–silica.

Another interesting phenomenon can be identified from Figure 4a,b. The gap between the *R*_t_ + *T*_t_ curves of the bare grating and nanostructured grating was more evident for the TM-polarized incidence. The schematic shown in Figure 5 can be used to explain this phenomenon. For an arbitrary TE-polarized light wave passing through the SWS layer, the electric field is always perpendicular to the cone-shaped pillars. However, for an arbitrary TM-polarized light wave, the field can be decomposed into two components, one perpendicular to the nanopillars and the other parallel to them. Therefore, the interaction of the TM-polarized light wave with the SWSs was stronger than that of the TE-polarized light wave, resulting in a stronger scattering for the TM-polarized light.

## 3. Conclusions

In summary, we have proposed and demonstrated the applicability of self-masking RIE to fabricate antireflective SWSs on fused silica transmission grating surfaces. Random cone-shaped nanopillars with an average height of 300 nm and bottom width of 200 nm were successfully fabricated on the grating surfaces. The SWSs exhibited excellent antireflection performance, where the reflectance from the grating surface was suppressed to zero over a wide range of incident angles. Although the light scattering on the disordered SWSs caused some losses, which can be avoided with smaller nanostructures and longer working wavelengths, the obtained transmission improvement was impressive. We also revealed that the duty cycle of the grating might change during RIE treatment in the case of oblique lateral walls in the grooves, which could lead to alterations of the diffraction orders. This could be utilized to provide more design flexibilities of diffraction gratings. Considering the easy operation, low cost, and effectiveness of the one-step self-masking RIE technique, it can be a promising way to fabricate antireflective SWSs on transmission–diffraction optic elements.

## Figures and Tables

**Figure A1:**
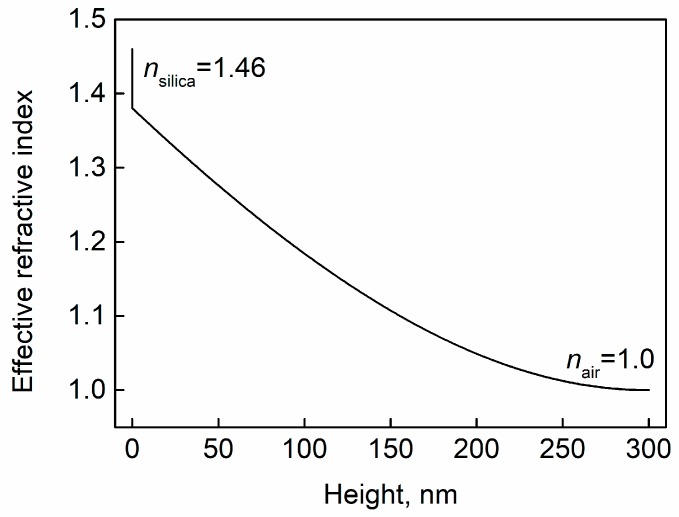
The effective refractive index profile versus subwavelength structure height calculated from Equation (A1) with the nominal conical nanograss model.

## Supplementary Materials

The following are available online at http://www.mdpi.com/2079-4991/9/2/180/s1, Figure S1: Schematic of the experimental setup for the measurement of the reflection and transmission efficiency with each individual diffraction order, Figure S2: Finite-difference time-domain model for nanostructured grating. (a) x–z view (vertical view); (b) 3D simulation model.

## Appendix A

Based on effective medium theory [34], the effective index for normal incidence can be expressed as:

*n*_eff_ = [*n*_air_^2^ × (1 − *f*) + *n*_silica_^2^ × *f*]^1/2^, (A1)

where *n*_eff_, *n*_air_, and *n*_silica_ are the effective, air and silica refractive indices respectively, and *f* is the nanostructure filling factor. Equation (A1) is accurate when the nanostructure size is much smaller than the wavelength, i.e., SWS. The SWSs shown in Figure 1 have a conical profile. Hence, we can calculate the effective reflective index using the close packed cone array model. We assume that the cone diameter and height are 200 nm 300 nm respectively according to the SEM images. By dividing the cone into 300 horizontal layers and calculating *f* for each layer, the variation of effective reflective index *n*_eff_ versus the cone height *H* from the substrate to the air can be obtained as Figure A1 shows, which indicates a gradual change of the effective refractive index between fused silica substrate and air.
