# Supplementary material for: Fabrication of Antireflective Nanostructures on a Transmission Grating Surface Using a One-Step Self-Masking Method
Source: Nanomaterials (Basel). 2019 Feb 1;9(2):180. doi: 10.3390/nano9020180 (PMC6410241; doi:10.3390/nano9020180)
Supplement: Supplementary file 1 [file nanomaterials-09-00180-s001.pdf]

Fabrication of Antireflective Nanostructures on a Transmission Grating Surface Using a One-Step Self-Masking Method

1. Experiments for Measuring the Reflection and Transmission Efficiency with Each Diffraction Order

The measurement of the reflection and transmission efficiency with each individual diffraction order was performed based on the experimental setup shown in Figure S1. The incident light with wavelength of 532 nm was generated from a diode-pumped solid-state continuous laser and passed through a polarized beam splitter (PBS) to control the polarization of the incident light, where the parallel light had TM polarization and the vertical light had TE polarization. When the TE polarization (s light) was measured, only mirror 1 was used, while when the TM polarization (p light) was measured, mirror 2 and mirror 3 were used. The tested grating was mounted on a rotating table to control the incident angle. The polarized light entranced the tested grating from its back side (i.e., the plane surface). Light power meters were used to measure the powers of the incident light, transmitted light and reflected light with each individual diffraction order respectively. Transmitted and reflected diffraction efficiencies were calculated from these measured powers respectively.

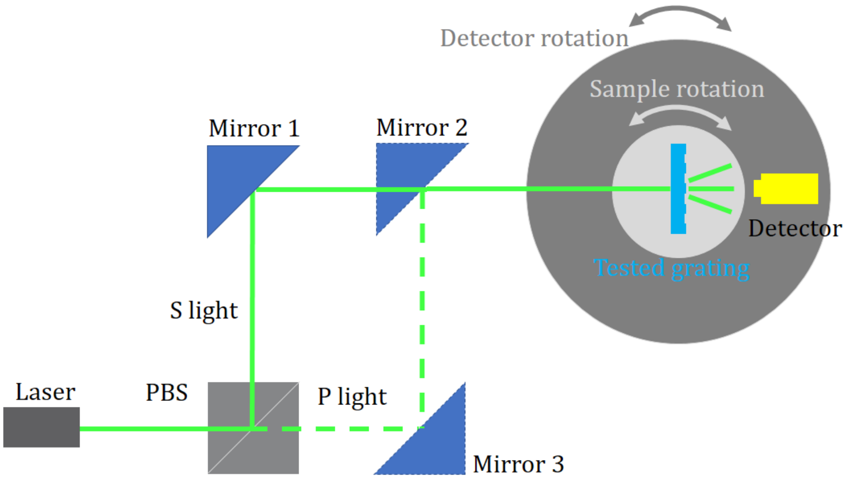

Figure S1. Schematic of the experimental setup for the measurement of the reflection and transmission efficiency with each individual diffraction order.

2. Detailed Information of the FDTD Simulations

The simulations in this paper are implemented by the three-dimensional finite-difference time-domain (3D-FDTD) algorithm with the commercial software package, FDTD solutions, provided by Lumerical Solutions, Inc. The schematic of simulation is shown in Figure S2. The simulation region was set as a cuboid of 3 μm × 0.4 μm × 5 μm volume, with periodic boundary conditions in x and z directions, and the perfectly matched layers (PMLs) boundary condition along the y direction. All the objects, sources, and monitors, were laid in this simulation volume. The incident light is set as plane wave propagating along the +y direction. Grid size is 10 nm × 10 nm × 10 nm. The simulated bare grating has the same geometrical parameters as those in Figure 1b, and the nanostructured grating had a new duty cycle of 0.65 and randomly-distributed nanocones with height of 300 nm, bottom

width of 200 nm and average period between nanocones as 200 nm. The optical constants were taken directly from the database of the FDTD software, where the refractive indexes of air and fused silica were 1 and 1.46 respectively.

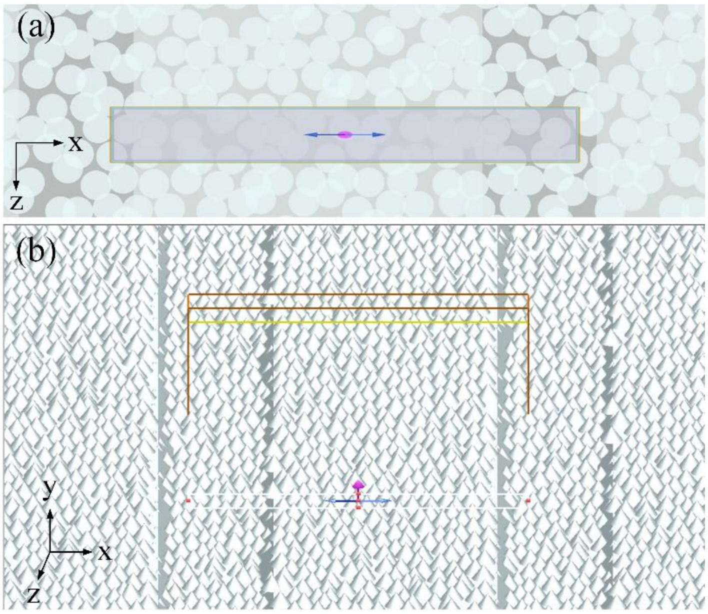

Figure S2. Finite-difference time-domain model for nanostructured grating. (**a**) x–z view (vertical view); (**b**) 3D simulation model.
